# Overcoming Barriers to Clinical Translation: MG1 Maraba Virus as an Emerging Platform for Oncolytic Immunotherapy

**DOI:** 10.3390/v18060617

**Published:** 2026-05-28

**Authors:** Tareq Abualfaraj

**Affiliations:** 1 Department of Basic Medical Sciences, College of Medicine, Taibah University, Madinah 42353, Saudi Arabia; tabualfaraj@taibahu.edu.sa; 2 Health and Life Research Center, Taibah University, Madinah 42353, Saudi Arabia

**Keywords:** Maraba virus, MG1 strain, oncolytic virotherapy, Rhabdoviridae, tumor microenvironment, cancer immunotherapy

## Abstract

Oncolytic viruses (OVs) exploit key hallmarks of cancer to selectively replicate in malignant cells, leading to tumor cell lysis, modulation of the tumor microenvironment, and induction of antitumor immunity. These viral platforms have been engineered to enhance tumor specificity, intratumoral spread, and immunotherapeutic efficacy. Among them, rhabdoviruses, particularly vesiculoviruses, have emerged as promising candidates due to their rapid replication, high titers, and amenability to genetic manipulation. Maraba virus, a recently identified vesiculovirus, is a single-stranded negative-sense RNA virus with a favorable safety profile and minimal pre-existing immunity in humans. It demonstrates selective tumor tropism partly through low-density lipoprotein receptor (LDLR)-mediated entry and impaired antiviral responses in cancer cells. Genetic engineering of the wild-type Maraba virus led to the development of the MG1 strain, characterized by enhanced tumor selectivity, increased replication capacity, and potent cytolytic activity. Preclinical studies have demonstrated its efficacy as a monotherapy, a cancer vaccine vector expressing tumor-associated antigens, and in combination with chemotherapy and immune checkpoint inhibitors. MG1 also reshapes the tumor microenvironment, converting immunologically “cold” tumors into “hot” tumors, thereby enhancing immune-mediated tumor clearance. Compared to vesicular stomatitis virus, Maraba virus exhibits improved safety and reduced neurovirulence while maintaining strong oncolytic potential. This review aims to comprehensively summarize the biological characteristics of the MG1 Maraba virus, its genetic development, mechanisms of action, and current preclinical and clinical applications as a novel oncolytic immunotherapeutic agent.

## 1. Introduction

Oncolytic viruses selectively exploit fundamental hallmarks of cancer to replicate within malignant cells, resulting in tumor cell lysis, modulation of the tumor microenvironment, and induction of in situ vaccination effects [1]. Various viral platforms have been engineered as oncolytic vectors with enhanced tumor specificity, improved intratumoral dissemination, and the capacity for therapeutic gene delivery, positioning them as targeted cancer Immunotherapeutics [2]. Oncolytic viral immunotherapy has emerged as a promising therapeutic modality for cancer patients, with several oncolytic viruses (OVs) advancing to late-stage clinical evaluation across multiple malignancies [3,4,5]. Evidence from both preclinical studies and clinical trials indicates that these agents, either as monotherapy or in combination with conventional cancer treatments, offer significant potential to improve therapeutic outcomes [6]. To augment OV-induced antitumor immunity, multiple strategies have been explored. These include genetic engineering of OVs to express immunostimulatory cytokines and the development of oncolytic vaccine approaches, whereby tumor-associated antigens are encoded within the viral genome [7].

To date, two oncolytic viruses have received regulatory approval for clinical use. H101, an E1B-deleted adenovirus serotype 5, was approved by the China Food and Drug Administration (CFDA) in 2005 for intratumoral treatment of head and neck cancer in combination with chemotherapy [8]. Subsequently, talimogene laherparepvec (T-VEC), a genetically modified herpes simplex virus type 1, was approved by both the U.S. Food and Drug Administration (FDA) and the European Medicines Agency (EMA) for the treatment of advanced melanoma [9]. Talimogene laherparepvec (Imlygic) is a herpes simplex virus type 1 (HSV-1) genetically engineered through deletion of ICP34.5 and ICP47, along with insertion of the human granulocyte–macrophage colony-stimulating factor (GM-CSF) gene. In 2015, it became the first oncolytic virus to receive approval from the U.S. Food and Drug Administration (FDA) for intratumoral administration in patients with unresectable malignant melanoma [10]. In addition, several other oncolytic viruses are currently under clinical investigation, including adenovirus, vaccinia virus, reovirus, and vesicular stomatitis virus (VSV), as well as related vesiculoviruses, all demonstrating promising antitumor activity.

A naturally attenuated VSV, a prototypical rhabdovirus, represents an attractive oncolytic platform due to its favorable safety profile and the absence of pre-existing neutralizing antibodies in humans [11,12]. Among these, Maraba virus has recently emerged as a potent oncolytic candidate, having been first developed for this purpose in 2010. Maraba virus is an enveloped, single-stranded, negative-sense RNA virus belonging to the vesiculovirus genus within the Rhabdoviridae family [3]. Genetic modification of the wild-type virus led to the development of the MG1 strain, which exhibits enhanced tumor-selective replication and increased cytolytic activity [13]. Preclinical studies have demonstrated the efficacy of MG1 as a monotherapy, as a cancer vaccine vector expressing tumor-associated or viral antigens, and in combination with standard chemotherapeutic agents, as well as with immune checkpoint inhibitors in the neoadjuvant setting [4,14,15]. This current review highlights the biological characteristics of the MG1 Maraba virus, its development and applications, and its emerging role in oncolytic virotherapy across various malignancies in preclinical trials.

## 2. Biological Features of Maraba Virus

There are more than 100 known rhabdoviruses currently classified into six genera: Vesiculovirus and Lyssavirus, which infect mammals; Ephemerovirus, primarily affecting livestock; Novirhabdovirus, which infects fish; and Cytorhabdovirus and Nucleorhabdovirus, which infect plants and differ by cytoplasmic and nuclear replication, respectively, reflecting their broad host range and diverse biological behavior [13]. All are classically identified by their characteristic bullet-shaped virions. The archetypal members of this family include rabies virus and vesicular stomatitis virus (VSV), which are among the most extensively studied. Although these viruses share similar morphological features, they differ substantially in their life cycle, host range, and pathological effects [13]. These relatively simple viruses possess several characteristics that make them attractive as potential oncolytic agents. Vesiculoviruses are capable of infecting a wide spectrum of mammalian hosts (e.g., horses, cattle, pigs, mules, and rodents), where they typically cause a benign acute disease that manifests as oral vesicles or ulcers [13,16]. Many rhabdoviruses replicate rapidly and achieve high titers in mammalian cells. These combined properties significantly facilitate large-scale production and purification processes, which are often challenging in virus-based therapeutics. Consequently, Maraba virus is considered a nonpathogenic arbovirus and represents a promising candidate for therapeutic applications [17]. Furthermore, rhabdoviruses are highly amenable to genetic manipulation, allowing for transgene insertion, structure–function guided mutagenesis to enhance viral performance, and incorporation of reporter genes for in vivo tracking [13].

**Figure 1:**
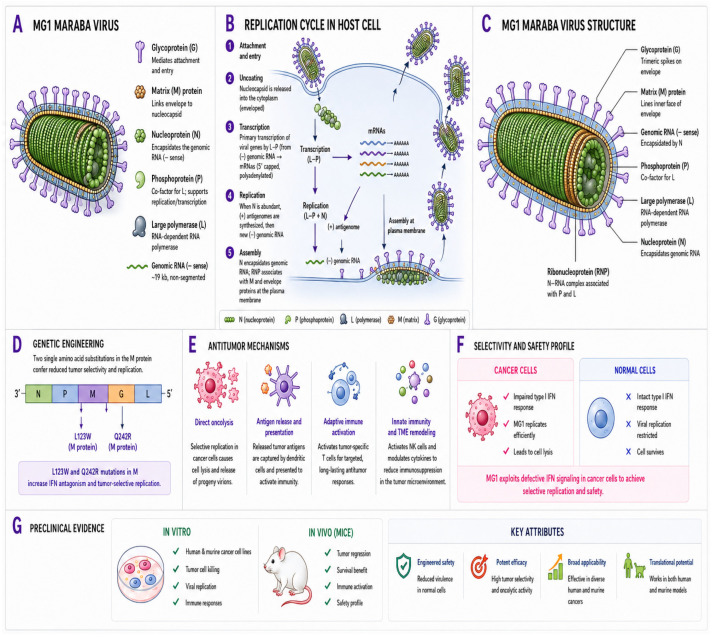
Schematic overview of the MG1 Maraba virus structure, genetic engineering, and mechanisms of antitumor activity. MG1 selectively infects cancer cells, undergoes replication, assembly, and oncolysis, releasing tumor antigens and progeny virions. This process stimulates innate and adaptive immunity, including NK and CD8^+^ T-cell activation, while remodeling the tumor microenvironment. Enhanced tumor selectivity and safety arise from impaired interferon responses in malignant cells.

Maraba virus, which has been recently characterized as a potent oncolytic virus, was first isolated from Amazonian phlebotomine sand flies in Brazil and has not been detected outside South America to date [17]. Phylogenetically, Maraba virus is an enveloped, single-stranded negative-sense RNA virus belonging to the vesiculovirus genus within the Rhabdoviridae family [18]. Both VSV and Maraba virus belong to this genus and share promising vaccine and oncolytic properties; however, they differ in biological behavior and therapeutic profile. Maraba virus demonstrates enhanced tumor selectivity and improved safety due to reduced replication in normal cells, whereas VSV exhibits broader tropism and a higher risk of neurovirulence [13]. The Maraba virion consists of a bullet-shaped enveloped particle (approximately 70 nm in diameter and 170 nm in length) that contains an approximately 11 kb single-stranded negative-sense RNA genome (NCBI reference: NC_025255). Its genome includes a 3′ leader sequence and a 5′ trailer sequence separated by five open reading frames encoding the nucleocapsid (N), phosphoprotein (P), matrix (M), glycoprotein (G), and polymerase (L) proteins (Figure 1).

Maraba virus utilizes, although not exclusively, the ubiquitous low-density lipoprotein receptor (LDLR) to enter target cells, which partly explains its broad tumor tropism [3]. LDLR is widely expressed across various tissues, including malignant cells, facilitating efficient viral attachment and internalization. However, additional entry pathways and receptor-independent mechanisms may also contribute to viral entry. Importantly, tumor cells frequently overexpress LDLR and exhibit impaired antiviral defenses, which further enhances viral uptake and replication, thereby supporting the selective infection and potent oncolytic activity of Maraba virus. LDLR facilitates viral entry by serving as an attachment receptor for viral glycoproteins, such as the G protein of vesiculoviruses [19]. Following binding, the virus–LDLR complex is internalized through clathrin-mediated endocytosis into endosomes. Progressive acidification of the endosomal compartment induces conformational changes in the viral glycoprotein, enabling fusion of the viral envelope with the endosomal membrane and release of the viral genome into the cytoplasm. Tumor cells often overexpress LDLR and exhibit impaired antiviral responses, enhancing viral uptake and replication.

## 3. Development of MG1 Maraba Virus for Tumor-Selective Oncolytic Immunotherapy

To enhance and selectively direct Maraba virus replication within malignant cells, the wild-type genome has been genetically engineered. Two-point mutations were introduced, resulting in L123W and Q242R substitutions within the matrix (M) and glycoprotein (G) sequences, respectively [13] (Figure 1). MG1 represents the newly engineered strain of Maraba virus and has demonstrated both oncotropic and cytotoxic activity across a variety of murine and human cell lines. It further exhibits selective tumor-killing capacity with minimal cytotoxic effects on normal cell lines [13]. MG1 has been applied in both in vitro and in vivo models to evaluate its therapeutic efficacy. In vitro, it is assessed in cell culture systems to examine tumor cell lysis, viral replication kinetics, and immune-related responses. In vivo, MG1 Maraba has been extensively investigated in animal models, particularly murine systems, to evaluate its effects on tumor regression, survival outcomes, and immune activation [4,14,15,20].

MG1 has been initially identified through systematic screening and is considered a novel engineered oncolytic virus specifically designed to selectively target and eradicate cancer cells. It demonstrates high tumor selectivity and strong cytolytic activity while preserving normal tissues. Notably, MG1 efficiently infects and eliminates both human and murine tumor cells, supporting its translational potential [13,20]. The engineered strain exhibits accelerated replication kinetics, increased burst size, and enhanced tumor cell killing compared with the wild-type virus and other Maraba mutants. In particular, Maraba virus displays broad oncotropism in vitro, and targeted genetic modifications have significantly improved tumor selectivity while reducing virulence in normal cells [20]. Within the tumor microenvironment (TME), MG1 Maraba induces a profound immunological shift, generating an inflammatory milieu that converts immunologically “cold” tumors into “hot” tumors, thereby enhancing susceptibility to immune-mediated attack [15,21,22]. It promotes oncolysis through the release of tumor-specific antigens, facilitating antigen spread and stimulating T cells capable of recognizing a broader spectrum of tumor-associated antigens [23,24]. Brun et al. identified Maraba virus as a highly potent oncolytic candidate characterized by robust tumor-killing activity, rapid replication, and high progeny yield [13]. Genomic and phylogenetic analyses have confirmed its classification as a vesiculovirus closely related to VSV, with the engineered strain demonstrating improved safety, a higher therapeutic index, and superior efficacy compared to VSV.

MG1 Maraba virus functions as an oncolytic therapeutic agent through both direct tumor targeting and the induction of antitumor immune responses, rather than acting solely as a vaccine platform. Its tumor selectivity is partly attributed to interferon (IFN)-dependent mechanisms, whereby viral replication is favored in malignant cells with impaired antiviral responses. This property allows MG1 to preferentially replicate within tumor cells while limiting replication in normal tissues [13]. In preclinical studies, MG1 has demonstrated potent antitumor activity through several complementary mechanisms, including direct tumor cell lysis, modulation of the TME, and induction of tumor antigen-specific T-cell responses [15]. Importantly, MG1 was capable of completing a full lytic cycle across a broad spectrum of human and murine cancer cell lines, underscoring its wide therapeutic applicability [13].

Beyond exploiting defects in type I interferon (IFN) signaling, MG1 Maraba virus mediates tumor cell killing through multiple interconnected mechanisms. It induces direct oncolysis by selectively replicating within cancer cells, leading to cellular destruction and release of progeny virions. This process promotes immunogenic cell death, facilitating the release of tumor-associated antigens and enhancing their presentation to the immune system. As a result, MG1 elicits adaptive immune responses, particularly through activation of tumor-specific CD8^+^ T cells [15]. In addition, it augments innate immune responses by activating natural killer (NK) cells, which target both infected and malignant cells [25,26,27]. Furthermore, MG1 modulates the TME by increasing pro-inflammatory cytokine production and reducing immunosuppressive signals, thereby converting tumors into a more immunologically active and responsive state [27].

## 4. Cellular Characterization of Host Cellular and Translational Responses During Oncolytic Maraba Virus Infection

Characterization of cellular responses during oncolytic Maraba virus infection reflects the interaction between viral replication and host translational control. Viruses depend on host cellular machinery for protein synthesis despite encoding their own transcriptional components. In response to infection, host cells often induce a global translational shutdown as an antiviral defense, primarily through disruption of cap-dependent translation pathways involving eIF4E and eIF2α [28,29,30,31,32,33,34]. This host response can limit protein synthesis; however, successful viral replication requires partial evasion or modulation of these inhibitory mechanisms. Phosphorylation of eIF2α by protein kinase R (PKR), which is activated upon detection of viral double-stranded RNA, suppresses ternary complex formation and promotes the assembly of stress granules, ultimately limiting viral replication [35,36]. Nevertheless, viruses can evade these host defenses by inhibiting stress granule formation or by employing alternative translation factors, such as eIF5B, to maintain protein synthesis under stress conditions [37,38]. Host cells initiate global translational shutdown as an antiviral defense mechanism during viral infection. These stress-adaptive responses may still permit selective synthesis of survival- or apoptosis-related proteins under conditions of translational suppression [39,40]. Such host translational alterations influence the cellular environment encountered during Maraba virus infection and may contribute to the balance between antiviral responses and efficient viral replication in malignant cells. Hassanzadeh et al. demonstrated that MG1 Maraba virus regulates host translational machinery through modulation of 4E-BP1 and eIF2α, leading to global translational suppression while selectively promoting the synthesis of pro-survival proteins such as Bcl-xL [18]. Despite activation of antiviral stress pathways, this mechanism supports host cell viability and enhances viral propagation. Notably, eIF5B was identified as a compensatory factor that enables translation in the absence of functional eIF2α, highlighting a non-canonical mechanism that sustains viral replication and defines key cellular features associated with Maraba virus infection.

## 5. Immunomodulatory and Antitumor Activity of MG1 Maraba in Oncolytic Viral Therapy

In addition to its potent oncolytic effects, the therapeutic efficacy of MG1 Maraba virus is strongly dependent on its inherent capacity to induce robust antitumor immune responses. Breitbach et al. reported early-phase clinical trials of MG1 Maraba oncolytic viral immunotherapy, highlighting mechanistic validation in parallel with safety evaluation [41]. The therapeutic approach focused on modulation of the TME, induction of antigen-specific T-cell responses, and verification of viral replication. Serial tumor biopsies and peripheral blood analyses, including gene expression profiling, immunohistochemistry, intracellular cytokine staining (ICS), and ELISpot assays, were utilized to assess immune activation, while molecular assays were employed to detect viral persistence and replication. Breitbach et al. demonstrated effective TME remodeling, increased infiltration of cytotoxic T cells, and measurable viral kinetics [41]. Although not statistically powered to determine efficacy, the study provided essential translational insights, supporting immune-mediated antitumor activity and informing the design of subsequent large-scale clinical trials.

Pol et al. further characterized Maraba virus as a potent oncolytic platform and developed an attenuated MG1 strain engineered to express a melanoma-associated antigen [20]. In murine models, MG1 alone was insufficient to induce a strong adaptive antitumor immune response. However, when incorporated into a heterologous prime–boost vaccination strategy, MG1 served effectively as a boosting vector, rapidly generating strong antigen-specific T-cell responses. In syngeneic melanoma-bearing mice, this approach significantly extended median survival and resulted in complete remission in more than 20% of treated animals [20]. These findings emphasize the potential of MG1 as a cancer immunotherapy vector with dual functionality, combining direct oncolytic activity with potent enhancement of adaptive antitumor immunity.

This capacity to stimulate antitumor immune responses is particularly relevant in the neoadjuvant setting [4]. Preoperative administration of MG1, whether replication-competent or attenuated, was shown to prevent adverse outcomes by sustaining antineoplastic immune pressure. Table 1 summarizes all preclinical studies evaluating MG1 Maraba virus as an oncolytic viral therapy across various solid tumor malignancies.

## 6. MG1 Maraba Oncolytic Virus Induces Direct and NK Cell-Mediated Cytotoxicity in Sarcomas, Including Ewing Sarcoma

Sarcomas are rare and heterogeneous malignant tumors of mesenchymal origin, in contrast to epithelial carcinomas, and are commonly treated with multimodal therapy involving surgical resection, chemotherapy, and radiation [47]. Immunotherapy represents a potential alternative to conventional chemotherapy; however, there has been limited focus on its application in sarcoma. Oncolytic viruses provide a novel strategy for enhancing immune responses against these difficult-to-treat malignancies. In contrast to carcinoma, only several preclinical studies have investigated the efficacy of oncolytic viral therapies in sarcoma models [14,48]. Nevertheless, few oncolytic viruses have demonstrated the ability to effectively infect and destroy human sarcoma cells in vitro [48,49,50]. In animal models, treatment with oncolytic viruses has been shown to delay the progression of sarcoma. Le Boeuf et al. emphasized the consistently poor prognosis associated with advanced bone and soft-tissue sarcomas, highlighting the urgent need for innovative therapeutic approaches [14]. In their study, four clinically relevant oncolytic viruses—Reovirus, vaccinia virus, herpes simplex virus (HSV), and rhabdovirus—were evaluated across in vitro, ex vivo, and in vivo experimental systems. Among these, MG1 Maraba virus demonstrated the greatest cytotoxic potency in vitro and achieved infection of more than 80% of human sarcoma tissues in ex vivo analyses [14]. Furthermore, in murine models, MG1 significantly enhanced long-term survival and led to durable tumor regression. Importantly, treatment with MG1 also induced a protective memory immune response against subsequent tumor rechallenge, supporting its potential as a promising candidate for sarcoma immunotherapy as well as for integration into combination therapeutic strategies [14].

Ewing sarcoma (EWS) has also been investigated as a potential target for treatment with OV therapies. EWS, first described by James Ewing in 1921, is an aggressive primary bone malignancy that predominantly affects children and young adults [51]. It is characterized by recurrent chromosomal translocations involving the *FET* and *ETS* gene families, resulting in fusion oncoproteins that drive tumorigenesis through transcriptional dysregulation [51,52]. Despite significant advances in oncology, therapeutic strategies for EWS have remained largely unchanged and continue to rely on multimodal approaches, including chemotherapy, surgical resection, and radiotherapy [53,54]. While patients with localized disease generally have a relatively favorable prognosis, clinical outcomes remain poor in cases of metastatic or relapsed disease [55]. Although immune checkpoint inhibitors have revolutionized cancer treatment, their effectiveness in EWS is limited, largely due to the tumor’s low mutational burden and insufficient neoantigen-driven immune responses [56,57,58]. However, the presence of cytotoxic immune cells, including CD8^+^ T lymphocytes and NK cells, within the TME has been associated with improved clinical outcomes, indicating a potential role for alternative immunotherapeutic strategies [25,26,59].

Oncolytic viruses have the capacity to remodel the TME into a pro-inflammatory state, thereby enhancing immune cell infiltration and reducing immunosuppressive signaling in EWS [5]. Tumor selectivity of OVs is partly explained by impaired antiviral defense mechanisms in malignant cells, which permit viral replication, whereas normal cells retain intact interferon-mediated responses that limit infection [60,61]. Importantly, OV-induced type I interferon signaling promotes activation of NK cells, enabling efficient elimination of both infected and malignant cells [62,63]. The engineered MG1 strain of Maraba virus demonstrates enhanced tumor selectivity and potent oncolytic activity, including efficacy in resistant and patient-derived tumor models, while also promoting NK cell-mediated cytotoxicity, thereby supporting its therapeutic potential in EWS. Barr et al. evaluated the MG1 Maraba virus as a therapeutic approach for EWS [27]. Their study utilized multiple in vitro systems, including established cell lines, drug-resistant variants, spheroid cultures, and patient-derived tumor cells. MG1 Maraba exhibited selective replication within EWS cells and induced robust direct oncolysis. In contrast, normal mesenchymal stem cells were largely spared, primarily due to intact interferon-dependent antiviral mechanisms. Furthermore, MG1 stimulated peripheral blood mononuclear cells to produce type I interferon, which in turn enhanced NK cell-mediated cytotoxicity against tumor cells [27].

## 7. Therapeutic Impact of MG1 Maraba Oncolytic Virus in Melanoma

OV therapies have demonstrated considerable promise in melanoma, a highly immunogenic malignancy that exhibits strong responsiveness to immune-based treatment strategies. These viruses selectively replicate within tumor cells, resulting in direct oncolysis and stimulation of antitumor immune responses. The most clinically advanced agent, and currently the only oncolytic virus approved worldwide for the local treatment of unresectable metastatic melanoma, is a genetically modified HSV (JS-1 strain), talimogene laherparepvec (T-Vec; IMLYGIC, Amgen; Thousand Oaks, CA, United States) [5]. In recent years, research efforts aimed at identifying additional OV therapies for melanoma have increased substantially.

MG1 Maraba virus has shown potent antitumor activity in melanoma models by enhancing tumor antigen presentation and inducing strong T-cell activation [20]. Moreover, MG1 Maraba virus has demonstrated the ability to modulate the tumor immune microenvironment and enhance therapeutic responses when combined with other immunotherapeutic strategies, supporting its potential as a treatment approach for advanced malignancies. The immune activation induced by oncolytic viruses has provided a strong rationale for combining virotherapy with immune checkpoint inhibitors. Clinically, this strategy has been evaluated using talimogene laherparepvec (T-Vec) in combination with pembrolizumab in patients with advanced melanoma in the MASTERKEY-265 (NCT02263508) trial. The Phase Ib portion of this study demonstrated that the combination therapy was well tolerated, with no dose-limiting toxicities observed [64]. Although MG1 Maraba virus therapy has not been extensively evaluated in clinical melanoma studies, Armstrong et al. investigated its immunological effects in melanoma models, focusing on tumor burden and TME immune dynamics [5]. Using both in vitro and in vivo experimental systems, they demonstrated that MG1 effectively primes cytotoxic T-cell responses against both tumor-associated and viral antigens [5]. High cure rates were observed in early-stage disease; however, therapeutic efficacy declined as tumor burden increased, which was associated with the development of a more immunologically “cold” tumor microenvironment. Importantly, combining MG1 with anti-PD-1 therapy restored antitumor responses in advanced disease, emphasizing the critical role of tumor burden in influencing responsiveness to OV.

## 8. MG1 Maraba Virus as a Successful Strategy for Breast Cancer Treatment

Breast cancer is a highly aggressive malignancy, with the majority of deaths attributable to metastatic disease occurring within the first three years following diagnosis [65]. The primary therapeutic approach, in addition to surgery, involves chemotherapy; however, certain tumor subtypes exhibit resistance to treatment, leading to poor clinical outcomes in these patients [66]. The current standard of care includes the administration of anthracyclines and/or taxanes following surgery to eliminate residual tumor cells [67].

A novel therapeutic strategy under investigation for metastatic breast cancer is the use of OV therapies. Paclitaxel (PAC) is a chemotherapeutic agent belonging to the taxane family that functions by stabilizing microtubules, thereby inhibiting cell division [68]. The combination of PAC with OV therapy has previously been evaluated using vaccinia virus and HSV in other disease contexts [69]. More recently, the MG1 Maraba virus has begun to play an important role in the treatment of advanced-stage breast cancer. A study conducted in 2016 explored this innovative therapeutic approach, particularly in cases characterized by chemotherapy resistance or disease recurrence [4]. The investigators assessed the combined use of MG1 Maraba virus and PAC across multiple murine breast cancer models. Both in vitro and in vivo analyses demonstrated that this combination was not only compatible but also synergistic, resulting in enhanced cytotoxic effects and increased viral replication within tumor cells [4]. Importantly, PAC did not adversely affect the safety profile of MG1. This combined therapeutic approach effectively controlled tumor growth and significantly prolonged survival in treated animals [4].

Furthermore, it was demonstrated that MG1 Maraba treatment promoted T-cell infiltration in the syngeneic 4T1 triple-negative breast cancer model [15]. Using a therapeutic model that closely reflects clinical practice, Bourgeois-Daigneault et al. administered OV therapy prior to surgical resection. Their results showed that early treatment effectively primed antitumor immunity and sensitized otherwise resistant tumors to immune checkpoint blockade [15]. This combined strategy prevented tumor relapse in the majority of treated animals and resulted in sustained and durable therapeutic benefit.

## 9. MG1 Maraba Virus as Personalized Immunotherapy in Leukemia

The immunogenic properties of MG1 have been leveraged to enhance the efficacy of cancer cell-based vaccines targeting leukemia and metastatic solid tumors in preclinical murine models [21,46]. Although Maraba virus demonstrated the ability to infect multiple leukemia and lymphoma cell lines in vitro, it did not exhibit significant therapeutic efficacy in mice bearing L1210 leukemia [21]. In contrast, administration of MG1-infected γ-irradiated leukemia cells (iLOV) resulted in complete responses in 60% of treated cases and provided full protection in prophylactic settings. Infection with MG1 effectively converted leukemia cells into a functional cancer vaccine, in part through the upregulation of key immunostimulatory molecules such as CD40 and OX40L [21]. This immunostimulatory effect was not reproduced by the use of Toll-like receptor (TLR) agonists or by noninfected irradiated leukemia cells. The process of Maraba-induced immunogenic cell death, characterized by enhanced antigen presentation and the release of pathogen-associated molecular patterns and damage-associated molecular patterns (PAMPs/DAMPs), played a central role in this response. These findings provide a strong rationale for further clinical investigation of autologous iLOV-based vaccine strategies.

## 10. MG1 Maraba Virus in Prostate Cancer

Prostate cancer (PCa) is estimated to have the second highest global incidence among male non-skin malignancies and represents the fifth leading cause of cancer-related mortality in men, underscoring the urgent need for novel therapeutic strategies [70]. MG1 Maraba is capable of infecting and killing a wide range of prostate tumor cell lines. Notable alterations in TME have been observed following administration of MG1 Maraba in murine models of PC (TRAMP-C2), which is classically considered an immunologically “cold” tumor type [43]. Treatment with MG1 Maraba resulted in tumors exhibiting upregulation of T-cell-associated markers, T-cell activation markers, immune checkpoint molecules, and genes involved in antigen processing, reflecting a transcriptional profile consistent with active T-cell-mediated immune responses and enhanced antigen presentation [43]. Through the induction of tumor antigen-specific T cells, modulation of the TME, and promotion of a pro-inflammatory state, MG1 Maraba may enhance the effectiveness of immunotherapeutic approaches such as ICIs, which depend on the presence of activated T cells within an inflammatory TME. These findings provide encouragement for combining MG1 Maraba therapy with anti-PD-1/PD-L1 immune checkpoint blockade strategies.

## 11. MG1 Maraba Virus and Natural Killer (NK) Cells to Kill Metastatic Tumors

NK cells are critical components of innate immunity, possessing a well-established capacity to recognize and eliminate tumor cells without the need for prior sensitization. Their antitumor activity has been consistently demonstrated across multiple OV platforms. Early investigations revealed that tumor regression following VSV therapy was dependent on both NK cells and CD8^+^ T lymphocytes [71,72,73]. Comparable observations were reported with reovirus and HSV, where NK cell infiltration and functional activity were essential determinants of therapeutic efficacy [74,75]. Additional evidence derived from the parapoxvirus study confirmed that OV administration induces robust NK cell activation and direct tumor cell lysis [76].

Importantly, even replication-deficient viral systems retained significant antitumor effects, emphasizing that immune activation—particularly NK cell-mediated cytotoxicity—rather than viral replication alone, represents a central mechanism underlying OV efficacy [77,78]. NK cells also play a pivotal role in controlling micrometastatic disease, especially in the perioperative setting, where impairment of NK cell function contributes to metastatic progression [79,80,81]. The MG1 Maraba virus further amplifies NK cell-mediated antitumor responses by promoting dendritic cell activation and cytokine production, thereby enhancing NK cell activation and improving targeting of metastatic tumor cells [45]. This effect is particularly relevant in postoperative contexts, where MG1 can restore NK cell function and reduce metastatic burden, supporting its role as a potent immunotherapeutic agent. Zhang et al. demonstrated that Maraba MG1 enhances NK cell-mediated antitumor activity through activation of conventional dendritic cells [45]. Both NK cells and dendritic cells were shown to be essential for achieving therapeutic efficacy. Although attenuated MG1 variants were also capable of inducing NK cell responses, maximal antimetastatic effects required administration at higher doses. Notably, while viral replication contributed to enhanced outcomes, it was not strictly required, as intact viral particles alone were sufficient to initiate NK cell activation. These findings support the potential of MG1 as a promising perioperative strategy aimed at reducing metastatic disease through augmentation of NK cell function.

## 12. MG1 Maraba Virus Is a Promising Therapy in Peritoneal Carcinomatosis

MG1-IL12-ICV was evaluated in immunocompetent murine models of peritoneal carcinomatosis using both B16 melanoma and CT26 colon carcinoma systems [21]. This approach resulted in a significant improvement in long-term survival in B16 tumor-bearing mice, with therapeutic efficacy shown to be dependent on NK cells and CD8^+^ T lymphocytes, as depletion of these immune populations completely abrogated the antitumor effect. Mechanistically, dendritic cell-derived CXCL10 was found to enhance NK cell migration and activation. These observations were further validated in human SW620 colorectal cancer cells co-cultured with patient-derived peripheral blood mononuclear cells (PBMCs). In CT26 models, repeated intraperitoneal administration of MG1-IL12-ICV achieved a 100% cure rate, in contrast to ≤40% observed with unarmed MG1-ICV. Notably, systemic co-administration of IL-12 alone did not yield similar efficacy, emphasizing the importance of localized cytokine delivery for achieving both safe and potent antitumor immune responses [21].

Tong et al. further investigated the cytotoxic activity of MG1 using a four-dimensional culture system that recapitulates the sequential stages of peritoneal carcinomatosis in ovarian cancer [44]. Remarkably, MG1 demonstrated the capacity to infect, replicate within, and induce cell death in ovarian cancer cells irrespective of their stage or structural organization, whether in adherent or suspended states, as individual cells or as multicellular spheroids. In addition, McGray et al. assessed a heterologous prime–boost vaccination strategy involving an initial vaccine followed by an antigen-armed oncolytic Maraba virus, combined with PD-1 checkpoint blockade, in a murine ovarian cancer model [42]. This combined approach elicited robust CD8^+^ T-cell responses and improved tumor control. However, immunosuppressive mechanisms within the TME limited overall efficacy, which was partially overcome through PD-1 inhibition, thereby enhancing therapeutic outcomes.

## 13. Clinical Translation and Ongoing Trials of the MG1-MAGEA3 Vaccine

To maximize the therapeutic efficacy of MG1, the virus has been engineered to express the tumor-associated antigen MAGE-A3, deploying it in a prime–boost vaccination strategy alongside a replication-deficient adenoviral vector. Comprehensive preclinical studies revealed that this Ad-MG1-MAGE-A3 regimen induced a massive expansion of MAGE-A3-specific CD4^+^ and CD8^+^ T-cells, generating robust and durable anti-tumor immunity without causing significant toxicity in non-human primates [82].

Based on these compelling findings and preclinical success, two major human clinical trials have been initiated to rigorously evaluate the safety, dosing, and efficacy of the MG1-MAGEA3 oncolytic vaccine in patients battling advanced solid tumors. The first study, a Phase I/II trial (NCT02285816), is primarily designed to establish the maximum tolerated dose and safety profile of the virus. This trial assesses MG1-MAGEA3 both as a monotherapy and in a prime–boost strategy using a replication-deficient adenovirus (Ad-MAGEA3) to pre-condition the immune system in patients with incurable, MAGE-A3-expressing metastatic malignancies [82]. Building on this prime–boost strategy, a subsequent ongoing Phase I/II trial (NCT02879760) is being conducted to specifically target non-small cell lung cancer in patients whose disease progressed after standard platinum-based chemotherapy or prior targeted immune treatments. This highly anticipated study combines the Ad-MAGEA3/MG1-MAGEA3 vaccine regimen with pembrolizumab, a widely used PD-1 immune checkpoint inhibitor [83]. Although extensive preclinical and in silico data strongly predict that combining MG1 with immune checkpoint inhibitors can successfully modify immunologically “cold” tumor microenvironments and overcome therapy resistance [5,84], current clinical results from these specific human trials have not yet been published. As of recent literature reviews, formal data regarding the primary efficacy endpoints and long-term clinical safety in human cohorts are still pending [83]. Therefore, while the preclinical results are highly encouraging, definitive conclusions on the clinical success of the MG1-MAGEA3 vaccine await the formal release of these trial outcomes.

## 14. Conclusions

Collectively, the evidence highlights MG1 Maraba virus as a versatile and potent oncolytic immunotherapy with dual mechanisms of direct tumor lysis and robust immune activation across multiple malignancies. Its ability to remodel the tumor microenvironment, enhance antigen presentation, and stimulate NK and T-cell-mediated responses emphasizes its therapeutic potential, particularly in resistant and advanced cancers. Preclinical studies consistently demonstrated improved survival and durable antitumor immunity, especially when combined with chemotherapy or immune checkpoint inhibitors. Future studies should prioritize clinical translation, optimization of combination regimens, and biomarker-driven patient selection to maximize therapeutic efficacy.

## Figures and Tables

**Table 1:** MG1 Maraba Oncolytic Virotherapy Studies Across Cancers.

	Year	Cancer Type	Models	Study Type	Key Findings	Reference
1	2025	Ewing Sarcoma	Cell lines, drug-resistant lines, spheroids, patient-derived cells, PBMCs	In vitro	Selective replication, strong oncolysis, IFN-dependent selectivity, enhanced NK-cell cytotoxicity	[27]
2	2024	Melanoma	Human and murine melanoma models	In vitro/In vivo	Strong T-cell priming, high efficacy in early tumors, restored response with anti–PD-1 in advanced disease	[5]
3	2020	Multiple cancers	Preclinical and translational data	Review	MG1 safety, tumor selectivity, immune activation, and clinical translation potential	[41]
4	2019	Ovarian Cancer	Murine models	In vivo	Boosted CD8^+^ T-cell responses; PD-1 blockade improved efficacy	[42]
5	2018	Triple-negative Breast Cancer	Murine TNBC models	In vivo	Sensitized tumors to checkpoint inhibitors, prevented relapse, induced durable responses	[15]
6	2018	Prostate Cancer	TRAMP-C2 mouse model	In vivo	Converted “cold” tumors to “hot”; increased T-cell infiltration	[43]
7	2018	Multiple cancers	Preclinical models	Review	MG1 as a potent oncolytic vaccine vector inducing antitumor immunity	[3]
8	2017	Sarcoma	Human/canine lines, tissues, murine models	In vitro/Ex vivo/In vivo	High cytotoxicity; infected >80% tissues; improved survival; immune memory	[14]
9	2017	Peritoneal carcinomatosis	B16, CT26, SW620 + PBMCs	In vivo/Ex vivo	NK/CD8^+^ dependent; MG1-IL12 achieved 100% cure in CT26	[21]
10	2016	Breast Cancer	EMT6, 4T1, E0771 models	In vitro/In vivo	MG1 + paclitaxel synergistic; enhanced replication; improved survival	[4]
11	2015	Ovarian Cancer	3D spheroid cultures	In vitro	Efficient infection and killing across stages; superior to other OVs	[44]
12	2014	Melanoma	Mouse models	In vivo	Strong T-cell responses; improved survival; partial remission	[20]
13	2014	Metastatic disease	Murine models	In vivo	Activated NK cells; reduced metastases; replication not essential	[45]
14	2013	Leukemia	Cell lines, L1210 model	In vitro/In vivo	iLOV vaccine induced 60% cure and full protection	[46]
15	2010	Multiple cancers	Tumor lines, mouse models	In vitro/In vivo	Identified MG1; strong selectivity and superior efficacy vs. VSV	[13]

## Data Availability

No new data were created or analyzed in this study.
